# Typical Marine Ecological Disasters in China Attributed to Marine Organisms and Their Significant Insights

**DOI:** 10.3390/biology13090678

**Published:** 2024-08-30

**Authors:** Lulu Yao, Peimin He, Zhangyi Xia, Jiye Li, Jinlin Liu

**Affiliations:** 1 College of Oceanography and Ecological Science, Shanghai Ocean University, Shanghai 201306, China; llyao166@163.com; 2 College of Ocean and Earth Science, Xiamen University, Xiamen 361102, China; xzy19028@163.com; 3 Key Laboratory of Ecological Prewarning of Bohai Sea of Ministry of Natural Resources, North China Sea Environmental Monitoring Center of State Oceanic Administration, Qingdao 266033, China; lijiye@ncs.mnr.gov.cn; 4 State Key Laboratory of Marine Geology, Tongji University, Shanghai 200092, China; 5 Project Management Office of China, National Scientific Seafloor Observatory, Tongji University, Shanghai 200092, China

**Keywords:** marine ecological disasters, Cnidaria, Annelida, Mollusca, Arthropoda, Echinodermata, harmful algal blooms, marine management

## Abstract

**Simple Summary:**

In China, in addition to green tides and red tides, outbreaks of Cnidaria (jellyfish organisms), Annelida (*Urechis unicinctus* Drasche, 1880), Mollusca (*Philine kinglipini* S. Tchang, 1934), Arthropoda (*Acetes chinensis* Hansen, 1919), and Echinodermata (Asteroidea organisms (commonly known as starfish), Ophiuroidea organisms, and *Acaudina molpadioides* Semper, 1867) not only damage marine resources, tourism, coastal industries, and navigation but also profoundly impact ecosystems near nuclear plants, beaches, and infrastructures, endangering human lives. This article suggests measures to mitigate future disasters, emphasizing enhanced monitoring and integration into China’s marine ecological disaster monitoring system. This review is conducive to enhancing researchers’ comprehension and reflection regarding biological disasters in marine ecosystems.

**Abstract:**

Owing to global climate change or the ever-more frequent human activities in the offshore areas, it is highly probable that an imbalance in the offshore ecosystem has been induced. However, the importance of maintaining and protecting marine ecosystems’ balance cannot be overstated. In recent years, various marine disasters have occurred frequently, such as harmful algal blooms (green tides and red tides), storm surge disasters, wave disasters, sea ice disasters, and tsunami disasters. Additionally, overpopulation of certain marine organisms (particularly marine faunas) has led to marine disasters, threatening both marine ecosystems and human safety. The marine ecological disaster monitoring system in China primarily focuses on monitoring and controlling the outbreak of green tides (mainly caused by outbreaks of some *Ulva* species) and red tides (mainly caused by outbreaks of some diatom and dinoflagellate species). Currently, there are outbreaks of Cnidaria (Hydrozoa and Scyphozoa organisms; outbreak species are frequently referred to as jellyfish), Annelida (*Urechis unicinctus* Drasche, 1880), Mollusca (*Philine kinglipini* S. Tchang, 1934), Arthropoda (*Acetes chinensis* Hansen, 1919), and Echinodermata (Asteroidea organisms, Ophiuroidea organisms, and *Acaudina molpadioides* Semper, 1867) in China. They not only cause significant damage to marine fisheries, tourism, coastal industries, and ship navigation but also have profound impacts on marine ecosystems, especially near nuclear power plants, sea bathing beaches, and infrastructures, posing threats to human lives. Therefore, this review provides a detailed introduction to the marine organisms (especially marine fauna species) causing marine biological disasters in China, the current outbreak situations, and the biological backgrounds of these outbreaks. This review also provides an analysis of the causes of these outbreaks. Furthermore, it presents future prospects for marine biological disasters, proposing corresponding measures and advocating for enhanced resource utilization and fundamental research. It is recommended that future efforts focus on improving the monitoring of marine biological disasters and integrating them into the marine ecological disaster monitoring system. The aim of this review is to offer reference information and constructive suggestions for enhancing future monitoring, early warning systems, and prevention efforts related to marine ecological disasters in support of the healthy development and stable operation of marine ecosystems.

## 1. Introduction

Currently, there is a global increase in frequent marine disasters, resulting in significant damage to nearshore and terrestrial ecosystems [1,2,3]. Simultaneously, these disasters disrupt fishing activities and tourism development, with some also causing destruction of residents’ livelihoods and productive resources, thereby impacting regional economic development [4,5,6,7]. For instance, storm surges and tsunamis [8,9] not only pose threats to human lives and property but also result in the destruction of marine and terrestrial habitats. This leads to the influx of land-based pollutants into the ocean or seawater encroaches on land areas. Consequently, freshwater resources essential for human survival become contaminated, exacerbating ecological crises in coastal regions and leading to the collapse and reorganization of social-ecological systems [10].

China is among the countries most affected by marine disasters [11,12], and these disasters are primarily attributed to physical and biological factors. The key disasters included in China’s marine disaster monitoring system encompass storm surge disasters, wave disasters, sea ice disasters, tsunami disasters, red tide events, and green tide events [13]. For instance, in 2023, the storm surge disaster (seven disaster incidents) resulted in the largest direct economic loss for China, totaling 2.4805027 billion yuan and accounting for 99% of the total direct economic loss from all disasters that year. Five wave disaster incidents led to a direct economic loss of 26.25 million yuan and caused eight fatalities or disappearances [13]. In the long term, both physical and biological factors can lead to significant indirect economic losses, particularly ecological and economic impacts.

**Figure 1:**
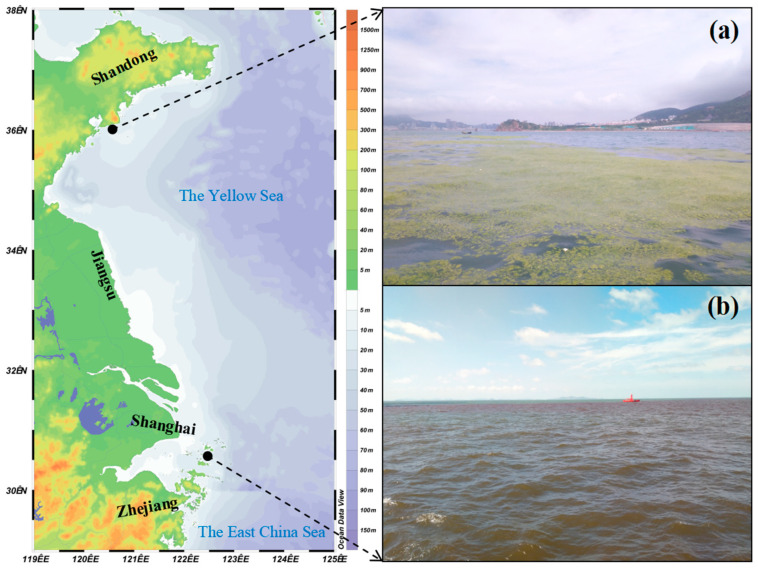
The large-scale outbreak of green tides and red tides occurred in China. (**a**) A green tide event occurred in the Yellow Sea in July 2019; (**b**) A red tide event occurred in the East China Sea in June 2019.

The continuous outbreaks of marine biological disasters in China have led to significant direct and indirect economic losses that cannot be overlooked. In recent years, there has been a frequent occurrence of green tides (Figure 1a) and red tides (Figure 1b), which have become the primary biological disasters monitored in China’s marine disaster monitoring system [14,15,16,17,18]. Red tides are characterized by sudden increases in population or aggregation of marine plankton (Figure 1b), resulting in water color changes (Figure 1b) or harm to other marine organisms [19]. In 2023, a total of 46 red tide blooms were discovered in China’s marine areas, with a total outbreak area of 1466 square kilometers; among them, 29 were toxic or harmful, covering an area of 1118 square kilometers; also, the East China Sea experienced the most occurrences and the largest total outbreak area [13]. Green tides exhibit large-scale outbreaks (Figure 1a), widespread distribution, and long duration. The Yellow Sea green tide outbreak is particularly typical, with macroalgae such as *Ulva prolifera* O.F. Müller, 1778 being the main species involved [18]. In 2023, the maximum covered area by the Yellow Sea green tide was recorded at 998 square kilometers, while its maximum distribution area reached 61,159 square kilometers [13], causing significant impacts and unquantifiable economic losses as well as algal prevention costs. Currently, the primary impact from marine disasters caused by biological factors (Figure 1) is on the loss to the marine ecological economy; nevertheless, it remains challenging to effectively assess this specific economic loss scale accurately. Objectively speaking, this issue requires sustained high-level attention along with improvements to disaster assessment standards.

The current China’s marine disaster monitoring system primarily focuses on microplankton and macroalgae [13]. However, there is minimal attention given to common marine animals that can cause ecological disasters, and they have not been included in China’s regular marine disaster monitoring system to date. The sustainable development of marine environmental protection and ecological systems is increasingly crucial for the development of coastal economies. The frequent outbreaks of jellyfish and starfish in the China Sea [20,21] have had significant negative impacts on fishery farming, ecological restoration, tourism, as well as the service functions of the marine ecological system. This has led to a series of economic and social problems, making it a long-term marine ecological disaster that urgently needs to be studied and solved in China. Additionally, small-scale outbreak phenomena are also being observed with *Acetes chinensis* Hansen, 1919; Ophiuroidea organisms; *Acaudina molpadioides* Semper, 1867; *Urechis unicinctus* Drasche, 1880; and *Philine kinglipini* S. Tchang, 1934 [22,23,24], gradually affecting human coastal infrastructure and the service functions of marine ecological systems. These phenomena have started attracting attention from researchers and the Chinese government.

Therefore, this review focuses on the fundamental biological background of the outbreaks of common marine animals—Cnidaria (jellyfish organisms), Annelida (*U. unicinctus*), Mollusca (*P. kinglipini*), Arthropoda (*A. chinensis*), and Echinodermata (Asteroidea organisms, Ophiuroidea organisms, and *A. molpadioides*) in China. Simultaneously, a detailed introduction of the current outbreak status of the aforementioned species in the China Sea is provided, with particular emphasis on summarizing and discussing the underlying causes of these outbreaks. The aim is to offer reference information and constructive suggestions for future disaster monitoring, early warning systems, and prevention efforts in order to support the healthy and stable development of the marine ecosystem.

## 2. Cnidaria—The Biological Background of Jellyfish and the Marine Ecological Disasters It Causes in China

Jellyfish, an important group of gelatinous plankton in the phylum Cnidaria, encompasses five categories: Hydromedusae, Siphonophore, Scyphomedusae, Cubomedusae, and Ctenophore [25]. Jellyfish originated in the Cambrian period, and jellyfish have survived five mass extinctions and continue to thrive in the global ocean [26]. Large jellyfish species are prevalent in the waters of China, Japan, and Korea [27]. Globally, more than 1400 species of jellyfish have been identified [28], with approximately 420 species reported in China’s nearshore waters [26,29]. Jellyfish possess a translucent and pliable body structure, characterized by a high water content and the absence of skeletal support [30]. They exhibit an alternation of generations in reproduction, and their demise can exert a substantial influence on biogeochemical processes and the cycling of life-source elements. Deceased jellyfish may serve as sustenance for micro- and small scavengers while also providing dissolved organic matter for microorganisms [31]. This influx of organic matter has the potential to impact plankton distribution and biomass, thereby playing a crucial role in maintaining marine ecosystem equilibrium and facilitating nutrient cycling.

Jellyfish are rich in minerals and proteins, with low fat, cholesterol, and calorie content, making them a natural delicacy in certain Asian countries [32]. The collagen present in jellyfish exhibits potential in preventing infectious pathogens and diseases and promoting health by eliciting immune responses [33,34]. Research has shown that collagen from *Nemopilema nomurai* Kishinouye, 1922, enhances immunity [35], while quniumucin extracted from various jellyfish also shows promising potential as a novel marine resource [33]. In the United States, salted and dried *Stomolophus meleagris* Agassiz, 1860 are utilized for the production of novel marine gelatin powders, serving as gelling agents, thickeners, and binders in food applications. They are increasingly recognized as a valuable source of collagen within the emerging fishery and food industry [32]. Owing to their extensive health benefits, jellyfish are progressively establishing themselves as an essential marine biological resource for nutritional supplements, cosmetics, and functional foods, demonstrating significant potential in both the food and health industries.

Surprisingly, jellyfish blooms have become a global phenomenon. Since 1995, numerous countries and regions, including the Baltic Sea, Japan, South Korea, India, Australia, the United States, Israel, and Scotland, have witnessed significant impacts from jellyfish outbreaks [36,37,38]. Jellyfish have proliferated and formed large aggregations, leading to blockages in the cooling water intakes of nuclear power plants. This has resulted in forced shutdowns of facilities such as the Madras Atomic Power Station in the Bay of Bengal and the Kashiwazaki Kariwa Nuclear Power Station in Japan. Similar incidents have also occurred at coastal power plants in South Korea, the United States, Israel, and Scotland [37]. Furthermore, jellyfish outbreaks have caused substantial economic losses within the fishing industry. For instance, Japan suffered losses of USD 20 million in 2003 and USD 250 million in 2005 due to *N. nomurai* outbreaks. Additionally, the Peruvian purse seiners of Ilo lost over USD 200,000 during 2008–2009 [38,39]. Moreover, jellyfish blooms have resulted in significant ecological harm, such as the incursion of Ctenophores along the Mediterranean coast of Spain and Israel in 2009, leading to extensive damage to the coastal ecosystem and fisheries [40]. In 2011, there was an outbreak of *Cephea cephea* Forskål, 1775, in the Red Sea, which served as an opportunistic food source for coral reef fish and disrupted the community structure of marine ecosystems [41]. In addition, approximately 52.8 million jellyfish stranded on Cabo Beach in 2012, with *Crambione mastigophora* Maas, 1903—a dominant species—exhibiting exceptionally high predation rates on bivalve larvae, potentially posing a serious threat to local oyster farming [42]. Moreover, a survey conducted among fishermen in the northern Adriatic Sea revealed that 70% of them had encountered jellyfish entanglement in their nets, resulting in reduced catches and economic losses amounting to up to 8.2 million euros per year [39].

**Figure 2:**
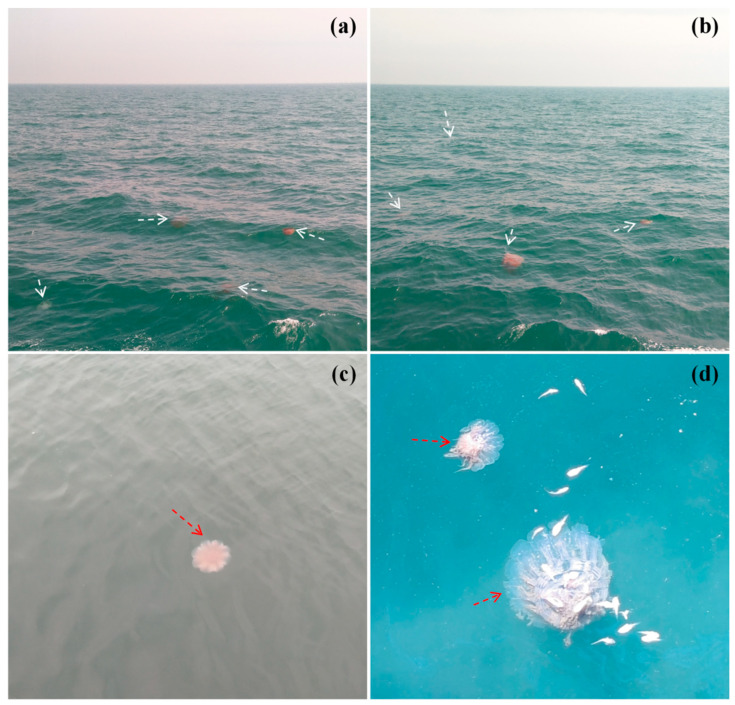
The outbreak of jellyfish occurred in the China Sea. On 21 June 2021, the jellyfish bloom was observed in the offshore waters of the Yellow Sea (**a**,**b**); on 26 June 2021 (**c**) and 3 June 2024 (**d**), the jellyfish bloom was observed in the East China Sea. The red or white dashed arrows indicate the location of the jellyfish that have been spotted.

In the Bohai Sea, Yellow Sea, and East China Sea (Figure 2), jellyfish outbreaks occur almost annually, leading to significant adverse effects on marine ecosystems and human livelihoods. Major species involved in these outbreaks include *N*. *nomurai*, *Aurelia aurita* Linnaeus, 1758, and *Cyanea nozakii* Kishinouye, 1891 [36]. Among these species, *N. nomurai* and *C. nozakii* primarily impact aquaculture and fishing by preying on the eggs or larvae of economically valuable fish and shrimp species [39]. This results in a decrease in fishing yields, leading to the obstruction or damage of fishing nets [43], thereby impacting fishing catches. Between 2000 and 2003, the annual catch volume in the northern East China Sea and the Yellow Sea’s fishery decreased by 64%, while the annual catch volume of *N. nomurai* increased by 250% [36]. In 2003, an outbreak of *Rhopilema esculentum* Kishinouye, 1891, occurred in the East China Sea, with an average biomass of 1555 kg/ha and a maximum biomass of 15,000 kg/ha; during this period, there was a reduction of about 20% in the catch volume of economically important species *Pseudosciaena polyactis* Bleeker, 1877. In 2004, an outbreak of *C*. *nozakii* occurred in Liaodong Bay, resulting in an approximately 80% reduction in edible *R. esculentum* and direct economic losses amounting to approximately USD 80 million [27,36].

*Aurelia aurita* has impacted the normal operation of some power plants in China. For instance, in August 2008, 20 to 50 tons of *A*. *aurita* were removed from the air intake screen at the Weihai coastal power plant [36]. In the same year, an outbreak of *A*. *aurita* blocked the rotary filter net at the seafront pumping station of the Qinhuangdao Power Company, leading to a unit shutdown and threatening power supply for Olympic venues [44]. Similarly, on 7–8 July 2010, over 10 tons of *A. aurita* obstructed the air intake screen at the Qingdao coastal power plant [36]. Additionally, in 2009, a large quantity of *A*. *aurita* blocked the seawater intake at Huadian Qingdao Power Generation Co., Ltd. (Qingdao, China), endangering its circulating water system. It took thirty employees over two days of continuous work to restore normal operations [45]. In addition, in 2013, an invasion by numerous jellyfish threatened safe operations at Liaoning Hongyanhe Nuclear Power Plant by blocking its cooling water intake [45]. Since 2014, the Hongyanhe Nuclear Power Plant has faced persistent challenges with jellyfish infestation at its intake, particularly from *N. nomurai* and *A. aurita*, posing a high risk of impacting the plant’s cooling source [46]. In 2014, an influx of jellyfish into the inlet area of the circulating water filtration system resulted in the shutdown of Units 1 and 2. Subsequently, in 2015, a significant population of jellyfish obstructed the intake due to breaches in both first and third barrier nets [37].

Furthermore, toxic jellyfish have increasingly appeared at coastal bathing areas, posing a threat to seaside tourism by endangering tourists. Species like *N. nomurai*, *C. nozakii*, *A. aurita*, *Physalia physalis* Linnaeus, 1758, *Pelagia noctiluca* Forsskål, 1775, and *R. esculentum* are notorious for their propensity to sting humans [36]. The stings from toxic jellyfish can result in symptoms such as skin redness, swelling, and burning pain, and in severe cases may lead to skin necrosis, cardiac and neurotoxic reactions, or even fatalities [36,45]. Since 1983, over 2000 incidents of jellyfish stings have been reported along China’s coast, resulting in 13 fatalities [44]. For example, in 2013 near Qinhuangdao City, *A. aurita* proliferated with a maximum density of 1500 × 10^4^ ind./km^2^, severely disrupting tourist activities and prohibiting swimming in the sea [44]. More than a thousand individuals sought medical attention due to jellyfish stings, which tragically resulted in the death of a child at Daihe Beach [36]. Meanwhile, in 2019, there were also jellyfish outbreaks reported in the vicinity of Haikou City in Hainan Province and near Maoming City in Guangdong Province [47].

Jellyfish outbreaks not only cause significant damage to marine fisheries, tourism, coastal industries, and ship navigation, but they also have a profound impact on marine ecosystems. The proliferation of jellyfish in large numbers leads to competition with other aquatic organisms for food, predation on marine organism larvae, and their stings can cause the mortality of certain marine organisms, resulting in a decline in ecosystem biodiversity [48]. Furthermore, the decomposition of jellyfish during their demise contributes to the accumulation of organic matter in the ocean, which promotes the rapid proliferation of toxic microorganisms. This process creates a low-oxygen zone at the ocean floor and releases acidic substances that harm marine organisms [49]. These impacts severely compromise the health of the marine environment and disrupt ecosystem stability.

The proliferation of jellyfish can be attributed to a combination of factors, including the impact of global climate change, alterations in environmental conditions, and human activities [50,51]. Firstly, global climate change has resulted in elevated sea temperatures, expanding the suitable temperature range for polyp budding and promoting their proliferation, consequently leading to increased jellyfish populations [30,49,51]. Climate change also induces variations in salinity, temperature, and other marine parameters that may influence jellyfish reproduction and distribution patterns. For instance, tropical jellyfish may migrate to subtropical and temperate regions due to these changes [52,53]. Secondly, environmental changes encompass diminished predator populations, overfishing practices, and water eutrophication. The decline in predators directly contributes to an upsurge in jellyfish numbers by reducing predation pressure and competition from other species [36]. Increased nutrient input leads to heightened plankton biomass, which serves as a food source for jellyfish. Simultaneously, eutrophication results in algae sedimentation and decomposition, causing oxygen depletion at the water column bottom—conditions unsuitable for many organisms but favorable for sustained jellyfish outbreaks due to their tolerance [54]. Furthermore, coastal nuclear power plants constructed by humans emit heat, potentially contributing to the increased survival rate of the polyp stage and the growth rate of the medusa stage of jellyfish [55]. Artificial structures in the ocean offer numerous attachment opportunities for the polyp stage of jellyfish, facilitating asexual reproduction through budding in certain jellyfish species [38]. Additionally, solid surfaces or shells (such as balanidae, Mytilidae, Vermetidae, and Hiatellidae) serve as attachment points for the polyp stage. The extensive cultivation of *Ruditapes philippinarum* A. Adams and Reeve, 1850, by human activities has altered subsea substrate and structure, providing ample attachment space and substrate for the polyp stage of jellyfish [56], thereby promoting jellyfish proliferation. Maritime shipping can introduce non-native species via ballast water and facilitate widespread dispersal of jellyfish [40]; a survey conducted in 2018 revealed that *A. aurita* reached peak populations in areas with high levels of human activity such as port construction and aquaculture in the Bohai Sea [52]. Moreover, the enforcement of China’s fishing ban system has led to a reduction in fishing activities and minimized human interference and mortality of jellyfish, thereby creating favorable conditions for the rapid proliferation of jellyfish [57]. Also, the surge in jellyfish populations may also represent an adaptive response to expand their distribution range and seek new habitats to cope with adverse environments [26].

## 3. Annelida—The Biological Background of *Urechis unicinctus* Drasche, 1880 and the Marine Ecological Disasters It Causes in China

**Figure 3:**
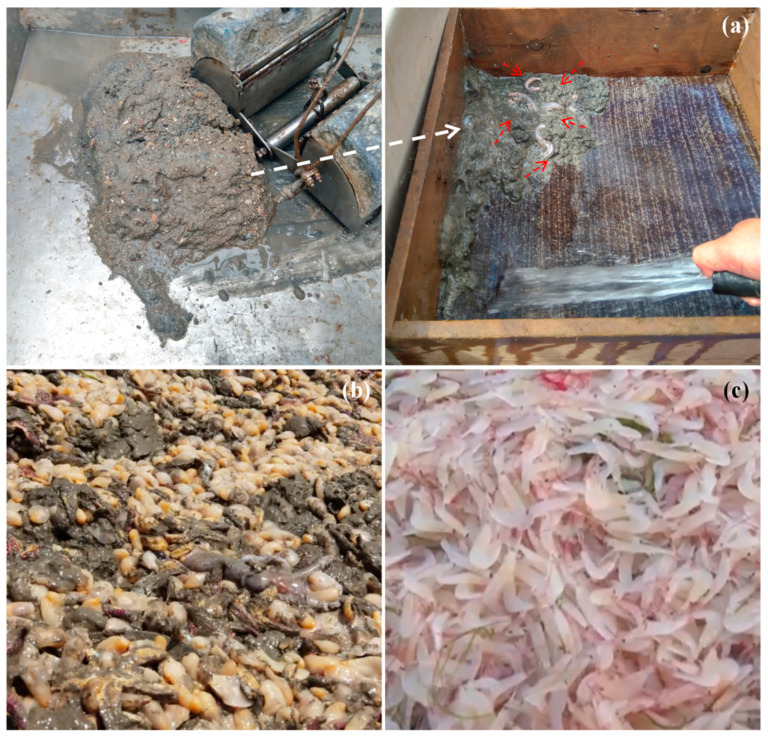
The outbreak of *Urechis unicinctus* Drasche, 1880; *Philine kinglipini* S. Tchang, 1934; and *Acetes chinensis* Hansen, 1919, occurred in China. *Urechis unicinctus* was discovered in the seafloor habitats of Jiaozhou Bay on 1 August 2019 (**a**); an outbreak of *P. kinglipini* occurred in the seawaters of Jiaozhou Bay in 2022 [89] (**b**); *Acetes chinensis* were captured in the Southern Yellow Sea in 2024 (**c**). Among them, the white dashed arrow indicates the sediment collected from the seafloor, and the red dashed arrow indicates *U. unicinctus*.

The *U. unicinctus* is commonly known as ‘Hai Chang’ in China and belongs to the phylum Annelida, class Echiurida, order Xenopneusta, family Urchidae, and was originally classified in the phylum Echiurioidea [58,59]. This species is widely distributed along the North Pacific coast, including the coasts of the Yellow Sea and Bohai Sea in China, Japan, the Korean Peninsula, and the Far East Region of Russia [60,61,62]. *Urechis unicinctus* typically inhabits muddy or sandy intertidal and sublittoral zones (Figure 3a) with ‘U’-shaped burrows and exhibits strong burrowing ability [63,64]. Its biological disturbance significantly enhances dissolved inorganic nitrogen (DIN) diffusion from sediments to water bodies by altering sediment structure through burrowing and feeding behavior [65,66], thereby promoting nutrient cycling in marine ecosystems [67]. Additionally, *U. unicinctus* feeds on phytoplankton, microzooplankton, and debris in water bodies to promote organic matter decomposition and transformation while reducing suspended particulate matter content. This contributes to purifying water quality and maintaining a clean and stable marine environment with significant ecological implications [68].

Although a small outbreak of *U. unicinctus* has only been reported in China, fortunately, *U. unicinctus* possesses significant potential for application in the fields of food and pharmaceuticals. Its meat is not only delectable but also abundant in protein and various essential amino acids [69], rendering it a high-value sea food with substantial market demand. Furthermore, its rich content of flavor amino acids can be utilized for the production of seafood seasonings [70,71,72]. Moreover, within the medical domain, *U. unicinctus* harbors diverse biologically active peptides and substances that exhibit anti-thrombotic, anti-tumor, antioxidant, antibacterial, and immune-regulatory properties [73,74]. The *U. unicinctus*-derived fibrinoclase demonstrates direct degradation of fibrin and fibrinogen while activating plasminogen without inducing acute bleeding side effects in animal models [75]. Additionally, antioxidant peptides VTSALVGPR, IGLGDEGLRR, and TKIRNEISDLNER isolated from the internal organs of *U. unicinctus* display promising antioxidant capabilities suitable for incorporation into health foods and beverages [76]. The active peptide DDL derived from *U. unicinctus* exhibits erectile function improvement properties [77]. Furthermore, glycosaminoglycan found in *U. unicinctus* manifests hypoglycemic effects by enhancing the body’s antioxidant capacity and tissue repair ability while increasing glucose tolerance and insulin sensitivity [78]. Lastly, due to its capability to tolerate and metabolize high concentrations of exogenous sulfides toxic to other organisms, *U. unicinctus* plays a crucial role in improving the quality of polluted sediments [63,73,79].

The recent widespread occurrence of *U. unicinctus* on the beaches of Yantai City, Shandong Province, China, has garnered significant attention. The reproduction and growth of *U. unicinctus* are seasonal, typically occurring in large numbers during spring and autumn with strong wind and wave conditions [63]. The large quantities of *U. unicinctus* washed onto the beach cover the sandy area, attracting residents and tourists to collect them; however, accidents can occur when individuals are swept into the sea by beachside winds and waves. Furthermore, due to its high tolerance for various heavy metals and easy accumulation in its body, consuming *U. unicinctus* in large quantities may pose a potential food safety issue [80,81]. It is noteworthy that *Listriolobus brevirostris* Chen and Yeh, 1958, which shares similar habits with *U. unicinctus*, has been found near multiple nuclear power plants, posing a threat to their safe operation as its *grappler method risk index* exceeds 50% [82,83]. Future massive outbreaks of *U. unicinctus* may clog seawater intake for nearby nuclear power plants, presenting a safety hazard.

Researchers generally believe that the significant outbreak of *U. unicinctus* is attributed to strong winds, turbulent seas, and extreme weather conditions. The sudden changes in sea temperature and disturbances in ocean sediments caused by extreme weather alter the habitat of *U. unicinctus*, compelling them to migrate from sedimentary bottoms to shallow waters and beaches. Additionally, the southward movement of a robust cold front brings about substantial cooling and strong northerly winds, potentially leading to the displacement of *U. unicinctus* onto the shore. Moreover, in Shandong Province’s Bajiao Bay, where the primary outbreak occurred, its trumpet-shaped geographical formation concentrates wave energy as they approach the coast. This, combined with high tides and strong winds, facilitates the stranding of *U. unicinctus* living on the seafloor. Furthermore, substrate conditions significantly impact their growth and reproduction; larvae exhibit higher survival rates in muddy environments compared with sandy ones [61,84]. Bajiao Bay’s bay-mouth has a gentle slope with flat beaches consisting mainly of silt-deposited layers conducive for *U. unicinctus’* development. The convergence water zone formed by the Yi River estuary provides abundant food for these organisms, while their mass stranding may also be linked to nocturnal breeding behavior [62]. Also, in recent years, local government initiatives releasing various marine species, including *U. unicinctus* larvae, have aimed at enhancing biological community structure and restoring marine resources but inadvertently led to rapid proliferation, disrupting local biodiversity and ecological equilibrium.

## 4. Mollusca—The Biological Background of *Philine kinglipini* S. Tchang, 1934, and the Marine Ecological Disasters It Causes in China

*Philine kinglipini*, commonly known as ‘Bai Ni Ma’ in China, belongs to the phylum Mollusca, class Gastropoda, subclass Opisthobranchia, order Cephalaspidea, and family Onchidiidae, with close genetic relationships to the family Aglajidae [23]. This species is mainly found in the Bohai Sea, Yellow Sea, and East China Sea, inhabiting sandy beaches in the lower layer of high tidal regions to muddy bottom substrates several tens of meters deep. As an omnivorous animal, *P. kinglipini* prefers feeding on cultivated mollusks such as clams and oysters. Its appearance resembles that of a snail, with a long oval-shaped shell that is thin and fragile with a white translucent color. Additionally, it has a well-developed radula and three differently sized rhomboid calcareous gizzards [23,85]. In terms of its ecological role, this species falls between nutritional levels II and III within the ecosystem [86,87,88].

Outbreaks of *P. kinglipini* occur only in China, with limited economic value, and its secreted slime can induce allergic reactions in humans. In recent years, outbreaks of *P. kinglipini* in the Qingdao Bay area of Shandong Province, China, have posed a significant threat to local shellfish aquaculture (Figure 3b) [89]. The species not only consumes large areas of bivalve larvae but also secretes slime, leading to localized oxygen depletion in the seabed and exacerbating shellfish mortality. In 2022, clam larvae in some aquaculture areas of Qingdao Bay decreased by two-thirds due to outbreaks of *P. kinglipini* and other species, resulting in losses of several million RMB for aquaculture farmers. Furthermore, biological surveys have revealed that *P. kinglipini* tends appears in high numbers in areas with abundant shellfish populations, such as its prevalence in Jiaozhou Bay [90,91], a frequency of up to 66.7% in the western Liaodong Bay [92], dominance on Nanjiu Island, which is primarily reserved for shellfish and other organisms [93], and an increasing abundance trend in the southern part of the Yellow Sea with densities reaching up to 55 ind/m^2^ [94]. *Philine kinglipini* exhibits a larger biomass in June in the sea off Yantai City, Shandong [95]; a survey conducted in 2022 revealed that *P. kinglipini* is the predominant species in the sea area near the Dao Island Archipelago in the Yellow Sea [96]. It is evident that *P. kinglipini* has exhibited significant breeding and dispersal patterns across multiple marine regions, potentially posing a threat to marine ecosystems.

The outbreak of *P. kinglipini* may be attributed to marine eutrophication, global climate change, and degradation of coastal environments, among other factors. In recent years, rapid human development and exploitation of marine resources have accelerated coastal degradation and disrupted the ecological balance in nearshore waters. The increased occurrence of marine biological outbreaks in China’s waters could be linked to starfish and jellyfish outbreaks that have disrupted the food chain balance [23]. Furthermore, global warming has favored the growth of temperature-tolerant *P. kinglipini*, while sedimentary mud deposits in certain areas also provide favorable conditions for its growth. The rapid expansion of shellfish aquaculture has provided a rich food source for *P. kinglipini*, accelerating its growth and reproductive capacity. Simultaneously, overfishing of economically important fish species has led to a decline in *P. kinglipini*’s predators (such as *Lateolabrax maculatus* McClelland, 1844, *Sebastes schlegelii* Hilgendorf, 1880, and *Hexagrammos otakii* Jordan and Starks, 1895) [97]. These combined effects have resulted in a significant increase in *P. kinglipini* populations, posing a threat to the stability of marine ecosystems and sustainable development of shellfish aquaculture.

## 5. Arthropoda—The Biological Background of *Acetes chinensis* Hansen, 1919, and the Marine Ecological Disasters It Causes in China

The species *A. chinensis*, belonging to Arthropoda, Malacostraca, Decapoda, and Sergestidae, *Acetes* [98], is a significant economic resource in China (Figure 3c) and is widely distributed in the near-shore waters of the Northwest Pacific region, including China, Japan, and South Korea [99]. Specifically, *A. chinensis* exhibits abundant resources in the coastal areas of the Bohai Sea, Yellow Sea, East China Sea, and northern South China Sea [100]. Its role as a primary consumer feeding on phytoplankton makes it crucial for marine ecosystems. Additionally, its significance as food for various marine organisms contributes to the material cycle and energy flow within marine environments [98,101,102].

*Acetes chinensis* is prone to outbreaks near China’s nuclear power plants, while it also holds considerable economic and nutritional significance. Its unique amino acid structure provides potential for the development of flavor substances, such as shrimp-flavored seafood seasonings [103]. Furthermore, *A. chinensis* is abundant in essential macronutrients and micronutrients, particularly vitamin B_5_, vitamin E, and calcium, making it suitable for use as a calcium supplement food and for the development of other health foods [104,105]. In terms of medicinal properties, the active peptide components in *A. chinensis* have demonstrated the ability to stimulate cellular and humoral immune systems for immune regulation enhancement [106]. Additionally, its neuraminidase inhibitory peptide exhibits anti-influenza virus effects and can serve as raw material for new anti-influenza drug development [107], while its ACE inhibitory peptide shows hypotensive effects [108,109,110].

However, *A. chinensis* poses a significant biological threat to the safety of cold source water for coastal nuclear power plants, as its ‘explosive’ aggregations have caused multiple blockages in intake screens. For instance, in January 2016, a large number of *A. chinensis* bloomed in the intake screen of the No. 2 reactor at Ling’ao Nuclear Power Plant, leading to filter blockage and cessation of the circulating water pump, resulting in an emergency shutdown [111,112]. In January 2019, there was an outbreak of *A. chinensis* near the intake screen of Daya Bay Nuclear Power Plant, with over 20 tons captured [113]; biological surveys revealed that *A. chinensis* was abundant in Daya Bay and accounted for over 99% of trawl catches near the nuclear power plant area, posing a threat to safe operations at Daya Bay Nuclear Power Plant [114]. In March 2020, the No. 4 reactor unit at Yangjiang Nuclear Power Plant was shut down due to massive blockage in the seawater circulation water filtration system by *A. chinensis*, which caused pump tripping and triggered a reactor emergency shutdown. After salvage and system restoration, the unit was reconnected to the grid the next day but experienced repeated failures due to subsequent ‘explosions’ of *A. chinensis* populations [111,115].

Multiple incidents of *A. chinensis* blockages in offshore nuclear power plants have been observed, primarily due to the combined effects of various factors. Firstly, in the context of global warming, changes in sea temperature directly impact the seasonal spawning migration and foraging activities of *A. chinensis* [101,114,116]. A significant drop in winter temperatures may force *A. chinensis* to migrate to warmer waters, and the vicinity of nuclear power plants often has higher water temperatures, increasing the likelihood of a large concentration of *A. chinensis* in the area [117]. Secondly, eutrophication and overfishing have disrupted the food chain. Human activities and meteorological factors such as storms, rainfall, and cold surges cause changes in the nutrient environment of the sea, leading to rapid changes in phytoplankton communities and extensive reproduction that provide abundant food resources for *A. chinensis* [117]. Simultaneously, overfishing has reduced predators of *A. chinensis*, further contributing to a potential population explosion. The construction of artificial reefs generates upwelling and counter-rotating currents that bring nutrients from the seabed to reach the sea surface, which may also affect distribution and population dynamics [102]. Additionally, dynamic changes in water flow patterns along with tidal movements sweep *A. chinensis* into nuclear power plant intake areas [114,115]. As more units are gradually put into operation at most nuclear power plant facilities sharing a common intake structure, it increases water flow rates near intakes, raising risks for ocean life being swept into them [118]. Due to *A. Chinensis*’s weak swimming ability and reproductive pattern closely tied to environmental conditions, causing sharp increases during specific seasons ultimately leads to biological blockage issues at nuclear power plant intakes [114].

## 6. Echinodermata

### 6.1. The Biological Background of Asteroidea and the Marine Ecological Disasters It Causes in China

The Asteroidea, the member of the Echinodermata phylum, is currently classified into five orders: Spinulosa, Forcipulata, Paxillosida, Valvatide, and Platyasterida [119,120,121]. There are approximately 1500 species worldwide [120,121,122], with over 100 species found in China; also, fossil remains of Asteroidea, Benthopectinidae species, have been discovered in Sichuan Province of China [123]. Meanwhile, Xiao et al. [124] identified nine new record species of Asteroidea in Chinese seawaters, including *Henricia arcystata* Fisher, 1917; *Henricia densispina* Sladen, 1878; *Henricia ohshimai* Hayashi, 1935; *Henricia exigua* Hayashi, 1940; and *Henricia pacifica* Hayashi, 1940, etc. [125].

Asteroidea, a carnivorous predator, inhabits sandy bottoms, muddy bottoms, rocky reefs, or coral reefs [126]. It plays a crucial role in regulating the population of its prey and maintaining local ecosystem equilibrium. Moreover, Asteroidea has the ability to absorb carbon from seawater for exoskeleton formation and, upon death, contribute to carbon sequestration in the ocean [121]. Additionally, it exhibits both sexual and asexual reproduction methods while possessing regenerative capabilities in its arms. In response to predation and environmental stressors, Asteroidea undergoes autotomy as an adaptive mechanism for survival and reproduction within dangerous habitats [120].

Asteroidea organisms are rich in lipids, proteins, polysaccharides, saponins, sterols, carotenoids, and cerebrosides, making them a promising source of functional health food processing ingredients [127]. They also contain a diverse range of pharmacologically active compounds, including polyunsaturated fatty acids with anti-hyperlipidemia, anti-hypertension, anti-platelet aggregation, and anti-thrombosis effects, as well as enhanced memory and improved autoimmune system disorders effects [128]. Furthermore, the asterosaponin of *Asterias rollestoni* Bell, 1881, exhibits significant inhibitory activity against various cancer cells [129]. Additionally, *Craspidaster hesperus* Muller and Troschel, 1840, *Stellaster equestris* Bruzelius, 1805, and *Asterina limboonkengi* G.A. Smith, 1927, have been traditionally used in Chinese folk medicine for treating goiter and rheumatism [122]. In conclusion, Asteroidea organisms demonstrate great potential for functional foods and pharmaceutical ingredients.

However, numerous marine ecosystems worldwide are under serious threat from biological outbreaks of Asteroidea. *Acanthaster planci* Linnaeus, 1758, has caused damage to coral reef ecosystems in the Indian Ocean and Pacific [130]. It is estimated that Asteroidea species have periodically erupted in the Great Barrier Reef for at least 7000 years [131]. Between 1985 and 2012, *A. planci* outbreaks resulted in a 42% mortality rate of corals in the Great Barrier Reef, leading to significant reductions in coral cover and biodiversity, as well as declines in fish density that feed on corals and structural and functional instability of the coral reef ecosystem [132,133,134]. Additionally, periodic outbreaks of *Asterias amurensis* Lutken, 1871, have affected nearshore waters of Australia, France, Japan, and Vietnam, posing threats to ecosystems while also causing damage to fisheries and marine aquaculture with substantial economic losses [132,135,136]. Furthermore, larvae of Asteroidea species can be transported globally through ocean currents and ship transport. For example, *A. amurensis* was transported to Tasmania, Australia, where it became an invasive species, causing significant damage to local fisheries and marine benthic ecosystems [121,135].

**Figure 4:**
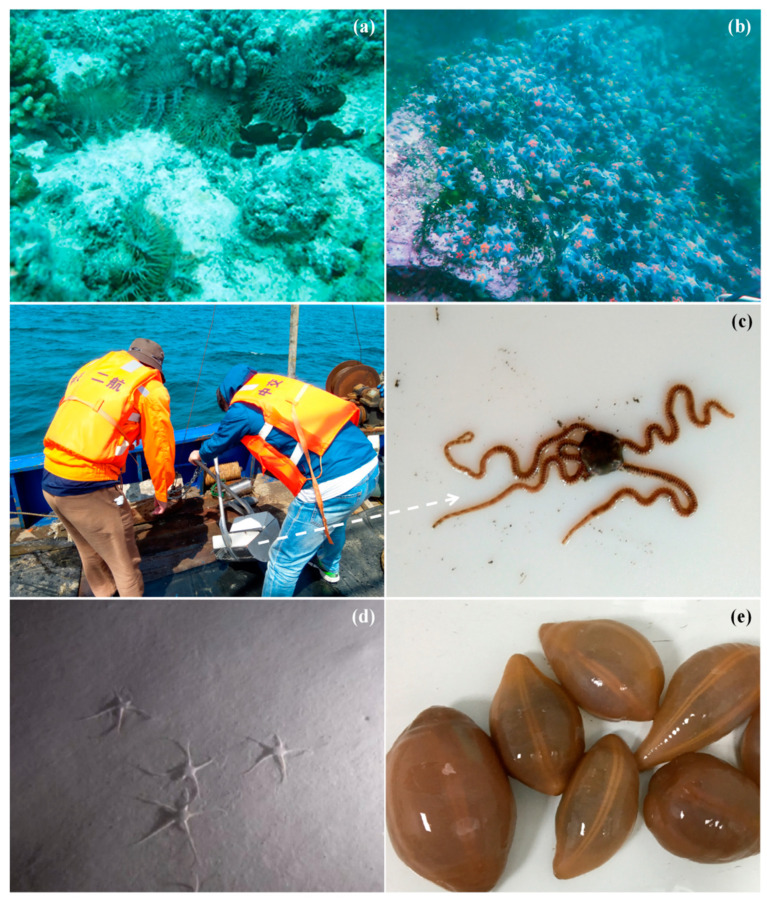
In 2018, an outbreak of *Acanthaster planci* Linnaeus, 1758, occurred in the sea area of Xisha Islands [140] (**a**) (Reproduced with permission; Copyright 2021; Science China Press); in 2021, an outbreak of *Asterina pectinifera* Muller and Troschel, 1842 took place in the sea area near Laopian Island, Dalian [126] (**b**); on June 22, 2019, the Ophiuroidea species was collected in the East China Sea (**c**); in May 2017, the population of Ophiuroidea species was observed in the sea area of north Yellow Sea [173]; (**d**) (Reproduced with permission; Copyright © 2019; Chinese Society for Oceanology and Limnology; Science Press and Springer-Verlag GmbH Germany; part of Springer Natures); and *Acaudina molpadioides* Semper, 1867 was collected from the East China Sea in 2018 [196] (**e**). The white dashed arrow indicates the Ophiuroidea species collected from the seafloor sediments.

In various marine regions of China, there have been multiple large-scale outbreaks of Asteroidea biological species (Figure 4a,b), with the outbreak numbers reaching 150,000–720,000 per hectare [137]. They are commonly referred to as ‘underwater locusts’ due to their significant population and destructive impact [138]. The highest concentrations of Asteroidea in China are found in the Yellow Sea, central Bohai Sea area, and the South China Sea. Among them, *A. amurensis* and *Asterina pectinifera* Muller and Troschel, 1842 mainly pose a threat to bivalve mollusks in marine aquaculture areas [126,138], while *A. planci*, *Acanthaster* cf. *Solaris,* and *Acanthaster solaris* Schreber, 1793 cause damage to coral reef ecosystems [139,140]. In marine aquaculture areas, the rapid increase in Asteroidea species leads to high density and large-scale aggregation, resulting in significant harm to fishery production. In 2006, the outbreak density of *A. amurensis* reached up to 300 ind/m^2^ in multiple aquaculture areas in Laoshan District and Huangdao District, Shandong Province, China [138], leading to a sharp decline in economically important species output; in 2007, the affected area within *R. philippinarum* aquaculture region in Jiaozhou Bay reached up to 60%, with a mortality rate as high as 80%, resulting in a loss of over 100,000 tons of output [138,141,142]; in 2008, an outbreak of *A. pectinifera* resulted in a 50% reduction in *Apostichopus japonicus* Selenka, 1867 production in the cultivation area [126]; in 2012, *A. amurensis* invaded the *Chlamys farreri* K.H. Jones and Preston, 1904 cultivation area in Qingdao City, leading to a decline of over 80% in *C. farreri* production [126]. In 2020, the average density of Asteroidea species outbreaks reached as high as 50 ind/m^2^ and peaked at 200 ind/m^2^ in the bottom-cultivated areas of *Crassostrea gigas* Thunberg, 1793, and *R. philippinarum* in Jiaozhou Bay, Shandong Province. This affected an area of approximately 66,666.7 hectares and caused direct economic losses exceeding 100 million RMB. Additionally, Asteroidea species outbreaks were observed in the waters of Jiaozhou Bay, Shandong Province, both in February and July 2022 [138].

In the marine areas of China where coral reefs thrive, the outbreak of *A. planci* exhibits a distinct periodicity. Its larvae consume phytoplankton, while adult *A. planci* can consume hundreds of square centimeters of coral reefs in a single night, causing severe damage to the structure and function of coral reefs and leading to a significant decline in biodiversity [143]. During the five-year period from 2006 to 2010, the outbreak of *A. planci* resulted in a decrease in coral coverage in the Xisha Islands from 60% to below 5%, resulting in the destruction of over 95% of live corals [134,136,140]. Between 2017 and 2021, the outbreak of *A. planci* led to a significant decline in coral coverage in the Nansha Islands, dropping from 33.0% to 0.9% [144,145,146]. In June 2019, the average density of *A.* cf. *solaris* in the northern South China Sea at Dongsha Atoll was recorded as high as 630 ind/ha [139]. By April 2021, the average density of *A.* cf. *solaris* in the central Nansha Islands had risen to as high as 630 ind/ha, with peak densities reaching up to 1920 ind/ha [139,144]. The destruction of coral reefs in the South China Sea will not only lead to a decline in biodiversity and deterioration of fishery resources but also pose a more serious threat by accelerating coastal reef erosion and endangering national security around China’s Xisha Islands and Nansha Islands, among others [142].

There are many factors that contribute to the mass outbreak of Asteroidea organisms, mainly influenced by the species’ growth characteristics, environmental conditions, predator numbers, and human activities. Asteroidea organisms have extremely strong reproductive capabilities, producing up to 65 million eggs per season [133] and up to 200 million eggs per year [145]. Additionally, global warming can cause an increase in sea temperature, which improves the survival rate of Asteroidea larvae and significantly increases the predation strength of adults [137,140,147]. Nutrient content in the sea may be increased through human activities, starfish excretion [148], and nutrients brought by storms or typhoons, further promoting the growth of phytoplankton and providing sufficient food and nutrition for Asteroidea larvae, thereby stimulating the growth and dispersal of the Asteroidea population [142,148,149]. Furthermore, overfishing of *Charonia tritonis* Linnaeus, 1758, *Chaetodon*, and Pomacentridae, Asteroidea’s predators, may increase the survival rate of Asteroidea larvae and adults, potentially leading to a massive outbreak of the species [130,150]. In addition, human activities such as the development of shellfish seeding technology and the expansion of aquaculture areas not only provide sufficient food for Asteroidea organisms but also destroy the original benthic marine ecosystem and increase the likelihood of Asteroidea outbreaks [120]. Meanwhile, in the management of marine resources, the ratio of the boundaries to the area of small marine protected areas is disproportionately large, which allows Asteroidea to enter protected areas rich in coral and leads to mass outbreaks, putting the increasingly fragmented and degraded coral reef ecosystem at greater risk [151].

### 6.2. The Biological Background of Ophiuroidea and the Marine Ecological Disasters It Causes in China

Ophiuroidea, a member of the phylum Echinodermata of invertebrate animals [152], is often misidentified by the general public as a starfish due to its morphological similarities with Asteroidea organisms. The current classification divides Ophiuroidea into five suborders: Ophiomyxina, Laemophiurina, Gnathophiurina, Chilophiurina, and Euryalidae. Originating in the Cambrian period 500 million years ago, Ophiuroidea experienced a mass extinction in the Permian period before gradually recovering in the Triassic period [153,154,155]. Most existing Ophiacanthidae organisms originated from deep sea environments, and species differentiation occurred no later than the end of the Triassic period [156]. There are at least 2064 globally recognized species of Ophiuroidea [155], with China alone hosting at least 211 species [154,155,157,158]. Fossils of Ophiuroidea have been discovered in inland or coastal provinces such as Hunan, Fujian, Guangdong, and Shaanxi, including *Ciliophiurina hunanensis* Lin, 1988; *Ophioderma huaanensis* Wu, 1980; *Ophioderma schistovertebrata* sp. nov.; *Ophiaulax bijieensis* sp. nov.; *Syntomospina kaiyangensis* sp. nov.; *Ophiolepis gulinensis* sp. nov.; and *Ophoderma qingchangensis* sp. nov. [159,160,161].

Recently, Chen et al. [162] have identified two new record species, *Ophiurothamnus discycla* H.L. Clark, 1911, and *Ophiurothamnus clausa* Lyman, 1878, on Huangyan Seamount in the central South China Sea; Li et al. [163] have discovered a new species named *Histampica haimaensis* sp. nov. in the cold seeps of the South China Sea; and Nethupul et al. [164,165,166] have found seven new species, including *Asteroschema domogranulatum* sp. nov., *Asteroschema shenhaiyongshii* sp. nov., *Ophiacantha aster* sp. nov., *Ophiomoeris petalis* sp. nov., *Ophiopristis shenhaiyongshii* sp. nov., and *Ophiophthalmus serratus* sp. nov. in the South China Sea, the seamount in the Northwest Pacific Ocean and the Mariana Trench. Additionally, recent years have seen discoveries of *Asteroschema* cf. *Bidwillae* McKnight, 2000; *Asteroschema rubrum* Lyman, 1879; *Asteroschema tubiferum* Matsumoto, 1911; *Asteroschema salix* Lyman, 1879; *Asteroschema* cf. *Lissum* H.L. Clark, 1939; and *Ophiotreta eximia* Koehler, 1904 [164,165,166]. These studies and discoveries further enrich our understanding of Ophiuroidea diversity and contribute to specimen preservation.

Ophiuroidea outbreaks mainly occur in China’s mariculture zones and waters near nuclear power plants. However, Ophiuroidea organisms (Figure 4c) play a crucial role in the economy, medicine, and environment, exerting a positive influence on human health and environmental sustainability. They act as primary or secondary consumers in the food chain by feeding on organic debris and small benthic organisms, thereby transferring energy to higher trophic levels. Additionally, they are significant contributors to the marine carbon cycle and nutrient cycling in sediments [158,167]. Due to their sensitivity to environmental changes, Ophiuroidea organisms can serve as indicators of global climate change and human activities’ impact. They can also be utilized for monitoring aquaculture activities’ effects on the marine environment and the risk of marine eutrophication [158]. Furthermore, Ophiuroidea organisms contain valuable compounds such as saponins, polysaccharides, and calcium with potential medicinal properties including anti-tumor, anti-viral, and anti-bacterial effects, along with anti-inflammatory properties. Moreover, they provide analgesic effects while enhancing calcium supplementation, immune system enhancement, and cardiovascular health. This provides abundant resources for research and development in the medical field [154,158,168,169].

However, the marine ecosystem is currently facing numerous challenges due to the impact of climate change and other factors, which in turn affect the ecological characteristics and population dynamics of Ophiuroidea organisms. Climate change has been shown to influence the reproductive activities of Ophiuroidea organisms [170]. Additionally, overfishing has resulted in a decline in the number of large carnivorous fish, leading to disruptions in the food chain and nutrient transmission at lower food chains. Furthermore, extensive expansion of marine aquaculture has caused significant damage to the marine benthic system, contributing to a rapid increase in Ophiuroidea populations [155]. In areas with abundant food sources, Ophiuroidea organism densities can reach up to 1200 individuals per square meter [158], potentially leading to sudden outbreaks and ecological disasters. Surveys have revealed that Ophiuroidea organisms are abundant in the Yellow Sea Cold Water Mass region [171,172] and have become dominant species in multiple aquaculture areas [173], posing a potential risk for outbreaks.

Additionally, *Amphioplus laevis* Lyman, 1874 presents a potential risk of obstructing the cooling systems of nuclear power plants in Fujian Province, Guangdong Province, and Hainan Province. Specifically, the *grappler method risk index* for blocking at Fuqing Nuclear Power Plant is 32.6%, while it is 45.8% for Daya Bay Nuclear Power Plant and Lingao Nuclear Power Plant, and 36.9% for Hainan Changjiang Nuclear Power Plant [174]. Ophiuroidea species pose a threat to the safe and stable operation of nuclear power plants. Furthermore, the Zhangzi Island Seafood Cultivation Base is the largest marine cultivation area for *Mizuhopecten yessoensis* Jay, 1857 in China; Ophiuroidea organisms (Figure 4d) are natural predators of *M. yessoensis*. The larvae of Ophiuroidea organisms, known as ophiopluteus, are abundant and easily dispersed, and the cultivated *M. yessoensis* in this area provides a rich food resource for their breeding and growth. Meanwhile, the increased numbers of *Ophiopholis mirabilis* Duncan, 1879, *Ophiura sarsii* subsp. *Vadicola* Djakonov, 1954, and *Siegophiura sladeni* Duncan, 1879, have led to clogging of fishing nets and underwater facilities, causing damage to fishing gear, reducing fishing efficiency, and resulting in economic loss [155]. This has further impacted the stability of benthic community structure and consequently affected the number and distribution of fishery resources.

Moreover, the alterations in the Yellow Sea Cold Water Mass impact the Ophiuroidea species, leading to their formation of high-density populations in the southern waters of the Zhangzi Island area during the summer season (Figure 4d). Among them, the predominant species, *O. sarsii,* preys on bivalve organisms such as *M. yessoensis* larvae [175], while *O. mirabilis* primarily feeds on suspended matter in the water, competing for food resources with *M. yessoensis* in its cultivated area [155]. In conclusion, the increase in Ophiuroidea species not only disrupts the stability of the benthic community but also poses a threat to economically significant species and impacts sustainable fishery resource development.

### 6.3. The Biological Background of Acaudina molpadioides Semper, 1867, and the Marine Ecological Disasters It Causes in China

*Acaudina molpadioides* (Figure 4e), a member of the phylum Echinodermata, class Holothuroidea, order Molpadida, and family Caudinidae [176,177], is closely related to the genus *Pseudostichopus* and shows some affinity with species in the suborder Dendrochirotida but not particularly close [178]. Widely distributed in subtropical waters of Bangladesh, Australia, Japan, the Philippines, and Indonesia [179,180,181], it mainly inhabits coastal areas of Hainan, Guangdong, Fujian, and Zhejiang Province in China [182,183]. *Acaudina molpadioides* commonly resides in burrows from intertidal zones to a depth of 80 m [184] and typically feeds on sediments beneath clayey silt layers. It is often found in deep water areas with higher organic carbon content in sediments [183], higher seawater salinity, and a lower temperature [182]. As a typical benthic organism, *A. molpadioides* excavates and consumes organic sediments to facilitate nutrient cycling within the detrital food chain. This profoundly alters sediment distribution and composition while playing an important role in modifying sediment structure and maintaining marine ecosystem stability.

The outbreak of *A. molpadioides* primarily occurred in the vicinity of nuclear power plants in China. However, *A. molpadioides* possesses high edible and medicinal value, as its body wall contains up to 89.16% crude protein [185], with a fat content of only 0.03% and almost no cholesterol [185]. Additionally, it is rich in mineral elements such as calcium, iron, zinc, and selenium [186,187,188], providing essential nutrients for the human body to enhance immunity and maintain good health. The polypeptide components of *A. molpadioides* exhibit antioxidant, anti-inflammatory, and hypoglycemic effects [185,186,189], effectively inhibiting α-amylase and Dipeptidyl peptidase-4 activity while increasing insulin secretion and reducing blood pressure by inhibiting Angiotensin-Converting Enzyme [190]. Various effective components in *A. molpadioides* can be used in medical drugs: AMC-2 improves non-alcoholic fatty liver disease by inhibiting Stearoyl-CoA Desaturase activity and damaging the synthesis of monounsaturated fatty acids in the liver [191]; collagen peptide has anti-infection and anti-tumor [192], physiological regulation functions along with hypotensive physiological functions that reduce oxidative stress to prevent acute liver injury [193]; sulfated polysaccharides have anti-inflammatory properties along with liver protection and antioxidant biological activities; saponins possess pharmacological functions including anti-tumor effects as well as antifungal properties alongside liver protection [186,189]. These active components indicate great potential for the development of *A. molpadioides* in both the food industry and medical fields.

Due to the ongoing deterioration of the global marine ecological environment and the escalating eutrophication in nearshore waters, there has been a proliferation and explosive aggregation of *A. molpadioides*. This phenomenon not only disrupts the stability of the benthic marine ecosystem but also poses a significant threat to the safe operation of coastal nuclear power plants. In August 2015, the Fujian Ningde nuclear power plant in China was forced to shut down due to blockage of the cooling system’s intake screen by *A. molpadioides*, causing a trip in the seawater circulation pump and triggering an emergency shutdown of the nuclear reactor. This directly impacted the safe operation of the nuclear power plant and posed a serious threat to nearby residents’ lives [176,182,184,194]. In Guangdong Province’s Daya Bay Navigation Channel area and southern region, *A. molpadioides* has formed a high-density population and is dominant for most of the year, with peak biomass reaching 170 g/m^2^. Assessments using the *grappler method risk index* have revealed that there is a 41.45% risk of blockage at Daya Bay Nuclear Power Plant caused by *A. molpadioides*, posing a safety threat to its stable operation [174,182]. Furthermore, investigations into large-scale benthic resources in the northern East China Sea indicate that *A. molpadioides* has consistently had the highest total biomass across all sampling areas and years. Excessive presence of *A. molpadioides* in Sanmen Bay has resulted in lower values for secondary production and production-to-biomass ratio (P/B), making it challenging to restore diversity and stability within this marine benthic ecosystem region [195].

The sudden and explosive aggregation of *A. molpadioides* in the sea area is the result of a complex interplay of factors. *Acaudina molpadioides* exhibits strong reproductive ability, with each female individual producing tens of thousands of egg cells. The environmental conditions, including the port pool and intake of the nuclear power plant, provide suitable habitat for *A. molpadioides* [184]. Under the influence of typhoons and other extreme weather conditions, as well as sea waves, seabed sediments are continuously stirred and accumulated, causing *A. molpadioides* larvae to enter the water body in a suspended state. Adult *A. molpadioides* migrate to shallow water areas to avoid clogged burrows. The weak adhesion ability of their body wall results in large quantities entering the water intake net of the nuclear power plant, forming high-density aggregation near the intake [182]. Furthermore, climate change will impact the reproductive patterns of sea cucumbers, with rising water temperatures promoting embryonic development. Additionally, eutrophication in the sea will lead to increased proliferation of planktonic plants, potentially forming harmful algal blooms. The subsequent sinking of dead organic debris from these plants will provide a substantial food source for *A. molpadioides* [183]. Moreover, sediment deposition and the erosion-reducing effects of nuclear power plant intake pipes and breakwaters may result in local sedimentation, further reducing competition for *A. molpadioides* in their habitat and benefiting their survival and reproduction [193]. It is important to note that due to their burrowing habits, limited mobility, large size, and uneven distribution, it is challenging to detect *A. molpadioides* in the ocean. This presents difficulties in monitoring and reducing the biomass of them during marine surveys [174,182].

## 7. Discussion

### 7.1. Short-Term Outbreaks of Marine Biological Disasters in China Are Anticipated to Persist

The impact of climate change on marine ecosystems is profound and multifaceted, resulting in elevated seawater temperatures and CO_2_ concentrations, ocean acidification, sea level rise, altered precipitation levels and oceanic structure, as well as heightened frequency of extreme weather events [197]. This impact directly influences the physiological processes and growth dynamics of marine organisms, shifts species distribution and community composition, alters interactions among species, and significantly impacts the biodiversity pattern in marine environments, posing a threat to the survival and stability of numerous species [198]. Moreover, the escalation of coastal resource development, global trade activities, and shipping operations has facilitated the introduction of non-native species through ballast water discharge [199]. This poses a risk to indigenous marine biological species while potentially reducing natural predators for explosively proliferating marine organisms. Concurrently, long-term challenges from widespread sources make marine pollution difficult to fully control [200,201]. Upon entering the oceanic environment, pollutants degrade water quality and substrate habitats, leading to diminished populations or even the extinction of sensitive species. Conversely resilient species with rapid reproduction rates can thrive in polluted settings, leading to dominance by a single species, which increases the likelihood of explosive population growth within marine biota, thereby exacerbating ecosystem instability. Consequently, it is anticipated that short-term occurrences of ecological disasters will persist within Chinese maritime territories.

Nuclear power plants have played a crucial role in meeting human energy demands, reducing reliance on fossil fuels, and mitigating greenhouse gas emissions [202,203]. However, the thermal discharges from their cooling systems have resulted in elevated water temperatures in the vicinity, creating optimal breeding conditions for certain organisms such as jellyfish and starfish. Furthermore, the disturbance of sediments around nuclear power plants has generated suitable habitats for specific benthic organisms like *A. molpadioides*, potentially leading to an upsurge in their population and disrupting ecological equilibrium. Due to the presence of protective barriers and fishing restrictions in the vicinity of nuclear power plants, certain species may experience reduced predation pressure in these areas, potentially leading to localized population surges that could impact the safe operation of the power plant. The sea areas adjacent to nuclear power plants are frequently challenging to obtain approval for conducting fishery fishing activities. Simultaneously, because of the biological enrichment effect in the vicinity of nuclear power plants, the nuclear radiation content in organisms might be relatively high, which will give rise to public apprehensions regarding the safety of related organisms in food and drug development. Consequently, the economic value derived from the outbreak organisms is considerably lower than the substantial economic losses resulting from the biological clogging accident in nuclear power plants.

China is a significant player in the development and operation of nuclear power plants [204]. Presently, there are 18 operational nuclear power plants in China, with a total of 58 generating units. According to geographical distribution, there are seven nuclear power plants located in the South China Sea, seven in the East China Sea, three in the Yellow Sea, and one in the Bohai Sea (Table 1). According to administrative divisions, Fujian Province hosts two nuclear power plants, Guangdong Province hosts four nuclear power plants, Guangxi Zhuang Autonomous Region hosts one nuclear power plant, Hainan Province hosts one nuclear power plant, Jiangsu Province hosts one nuclear power plant, Liaoning Province hosts one nuclear power plant, and Shandong Province hosts one nuclear power plant. Additionally, Taiwan Province has one nuclear power plant, and Zhejiang Province has five nuclear power plants (Table 1). Currently, at least five nuclear power plants nearby regions in China have experienced marine animal outbreaks [45,46,111,113,114,115,174,176,182,184,194], including Hongyanhe Nuclear Power Plant, Ling’ao Nuclear Power Plant, Daya Bay Nuclear Power Plant, Yangjiang Nuclear Power Plant, and Ningde Nuclear Power Plant, representing approximately 28% of China’s nuclear power facilities (Table 1). Nuclear power plants have the potential to replace some of the current mainstream thermal power generation, which is crucial for reducing carbon emissions. However, in recent years, marine fauna disasters have frequently occurred in nuclear power plant regions. This not only causes significant damage to the ecological environment but also has adverse effects on fisheries, tourism, public health, and industrial facilities and leads to public panic [20,21,36]. Therefore, it is imperative to urgently explore effective methods and management strategies for controlling the total biomass of explosion species of marine fauna near nuclear power plants to ensure their sustainable operation and harmonious coexistence with ecological systems.

In addition, typhoons and other natural disasters frequently transport marine organisms from their usual offshore habitats to the shore, leading to the phenomenon of marine organisms congregating in coastal areas [182]. Strictly speaking, this may not constitute a mass proliferation-induced outbreak. It is essential to conduct on-site surveys and assessments based on actual conditions to accurately differentiate the outbreak attributes. If biological species are driven towards the shore by external forces and form an aggregation phenomenon, this process represents only a temporary alteration in their distribution and does not signify an increase in population. Classifying it as a biological distribution anomaly caused by natural disasters is more appropriate. Accurately distinguishing between natural disaster-induced biological distribution anomalies and biological mass proliferation outbreaks is crucial for marine ecological research and management. The former typically has short-term effects with relatively limited long-term impact on ecological systems, while the latter may indicate underlying issues within ecological systems that require corresponding management measures for resolution. Although natural disaster-induced biological distribution anomalies do not qualify as biological outbreaks, they still warrant serious attention. These aggregated organisms may intensify local predation competition and temporarily exert pressure on coastal ecosystems. Furthermore, they serve as a reminder to further strengthen relevant research and monitoring efforts while enhancing our understanding of these complex ecological phenomena alongside acknowledging the profound impact of extreme weather events on marine ecosystems.

### 7.2. The Selected and Implemented Control Measures Should Aim to Minimize Disruption to Marine Ecosystems

The complexity of marine ecosystems and the dynamic changes in the environment pose challenges for predicting marine biological outbreaks. While monitoring and early warning technologies have developed, real-time coverage still has limitations, and the implementation of preventive measures is constrained by technical, economic, and policy factors. Consequently, the primary response strategy currently involves post-disaster management, primarily encompassing physical control, chemical control, and biological control. Presently, physical salvage stands as the predominant method of control; for instance, in 2008 off the coast of Weihai, 20–50 tons of *A. aurita* were salvaged [36]; in 2019 near the intake of Daya Bay Nuclear Power Plant [113], over 20 tons of *A. chinensis* were captured; while in a single day in 2021 at Jiaozhou Bay’s bottom-culture area [126], starfish weighing up to 50 tons were caught.

Fortunately, chemical control methods are seldom employed due to potential adverse effects such as water quality deterioration and non-target species damage. These methods can lead to water pollution and substrate environment destruction, which complicates their efficacy while impacting fishery resources. Similarly infrequent are regional biological control methods that require complex evaluations considering ecological relationships between target species and introduced ones along with adaptability assessments for potential risks associated with spread within local ecological systems. Furthermore, long-term environmental impact assessments are essential to prevent ecosystem imbalances resulting from introducing biological controls that could trigger outbreaks among other or introduced species. The application of biological controls necessitates stringent regulation and monitoring, ensuring sustainability while averting new ecological threats. Physical control methods generally offer greater environmental friendliness, albeit progress being slow with high labor costs, but minimizing benthic ecosystem disturbance results in relatively minor impacts on marine ecosystems overall healthiness. By reducing disturbances in benthic environments, nutrient release can be curtailed, thus preventing eutrophication occurrences alongside harmful algal bloom outbreaks, thereby safeguarding benthic organisms’ well-being as well as overall ecosystem integrity. Combining long-term monitoring scientific management technological innovation like developing more efficient fishing tools and improving operational methodologies will further optimize physical controls, enhancing efficiency and sustainability.

### 7.3. Effective Resource Utilization of Outbreak Species Is a Long-Term Solution

**Figure 5:**
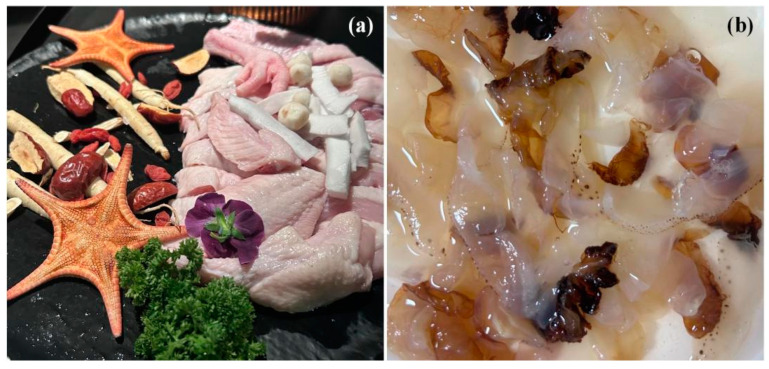
Due to their culinary appeal and distinctive flavor, starfish are frequently utilized as accompaniments in Chinese hotpot cuisine (**a**); some species of jellyfish have edible oral arms, which are commonly prepared as cold shredded jellyfish in China (**b**).

When marine organisms become the single or dominant species in a particular area and significantly impact human production and living activities, they are often classified as having the potential for a biological outbreak. From an objective standpoint, these outbreak marine organisms also represent high-quality biological resources, possessing both known and yet-to-be-developed edible, medicinal, and industrial values. It is imperative to develop effective pathways for utilizing these outbreak marine organisms (Figure 5); this approach can not only prevent the loss of biological resources but also rapidly reduce the biomass of marine organism waste while effectively controlling the scale of the outbreak in affected areas, thereby fostering advancements in the fishery industry [205,206]. Although the utilization of resources from outbreak organisms represents an approach to converting ‘disaster’ into ‘resource’, this utilization method is challenging to compensate for the extensive losses they inflict on the ecosystem and socio-economic domains. Hence, it can merely function as an ancillary method and cannot substitute for more fundamental ecological management and disaster prevention and control measures.

In response to the outbreaks of Cnidaria (jellyfish organisms), Annelida (*U. unicinctus*), Mollusca (*P. kinglipini*), Arthropoda (*A. chinensis*), and Echinodermata (Asteroidea organisms, Ophiuroidea organisms, and *A. molpadioides*) in China, three key issues must be addressed to further advance the sustainable utilization of these organisms. Firstly, it is imperative to establish effective storage methods for the collected or harvested outbreak species, necessitating a series of experimental simulations in product development, preservation techniques, drying processes, and crude product production. Additionally, formulation of execution standards for product production (e.g., food products) is essential. Secondly, there are limitations in the existing forms of products derived from outbreak species and an inadequate research foundation for certain outbreak species, such as *P. kinglipini*; their biological value remains incompletely explored. Thirdly, products derived from outbreak species require a comprehensive food supply chain to achieve long-term stability and development. This entails integration across upstream and downstream industries; only through the formation of stable market demand can resourceful utilization of outbreak species cease to be an issue.

### 7.4. Enhancing and Releasing Activities Require Careful Selection of Species to Be Released

At present, the implementation of aquatic organism enhancement and release activities necessitates prudence. Uninformed release practices may detrimentally impact the ecological environment. The execution of marine enhancement and release activities demands a robust scientific foundation and fundamental research. The Regulations on Aquatic Organism Enhancement and Release issued by the Ministry of Agriculture and Rural Affairs of the People’s Republic of China explicitly prohibit the utilization of foreign species, hybrid species, genetically modified species, or other species that do not align with ecological requirements for enhancement and release activities. It is imperative to conduct enhancement and release activities for endangered species, nationally protected wild animals, or significantly depleted fishery resources such as *Larimichthys crocea* Richardson, 1846, *Acipenser sinensis* Gray, 1835, *Trachidermus fasciatus* Heckel, 1837, *Hippocampus kelloggi* Jordan and Snyder, 1901, *Tridacna crocea* Lamarck, 1819, *Branchiostoma belcheri* Gray, 1847, and *Tachypleus tridentatus* Leach, 1819, in order to restore their population size and genetic diversity levels [207,208,209]. Nevertheless, detailed guidelines for enhancing and releasing economically important species are currently lacking.

Humans often prioritize the economic and ecological value of a select few species, neglecting the interconnectedness and holistic nature of ecological systems. For instance, the ongoing outbreak of golden tides in China caused by *Sargassum horneri* (Turner) C. Agardh, 1820, serves as a pertinent example [6,210]. In recent years, local governments have introduced a substantial number of *S. horneri* seedlings into nearshore waters in Zhejiang Province and Shandong Province to establish marine pastures. The precise origin of the current golden tide outbreak in China remains unknown, necessitating further research to ascertain whether the introduction of *S. horneri* has significantly contributed to this phenomenon. It is imperative to consider that artificially promoting the dominance of some particular species within a localized area may not represent an optimal solution for marine ecological restoration. The process of enhancing and releasing marine fauna should also carefully evaluate its potential impact on disrupting marine ecological balance. Notably, there has been a sudden proliferation of *U. unicinctus* along beaches in Yantai City, Shandong Province. This surge can be attributed to continuous releases by local government breeding programs involving numerous indigenous marine species, including *U. unicinctus* larvas; it is important to recognize that such activities may inadvertently facilitate the proliferation of ecologically adaptable species like *U. unicinctus* and disrupt the original ecological equilibrium.

In summary, the selection of species for enhancement and release activities should be conducted with careful consideration. It is imperative for scientists to prioritize understanding the holistic nature of the ocean and its inherent capacity for self-repair, particularly focusing on mitigating marine environmental pollution.

### 7.5. Further Enhance Fundamental Research to Ensure the Sustainable Development of Offshore Ecosystems

The frequent occurrences of marine ecological disasters present a significant threat to the sustainable development of marine ecosystems. Ensuring the sustainability of marine ecosystems is essential for maintaining ecological equilibrium, conserving biodiversity, fostering economic activities, and mitigating the impact of disasters. Various types of marine ecological disasters actually provide insights into the evolutionary processes shaping the structure and function of marine ecosystems and may even indicate changes in their structure.

To effectively address marine biological outbreaks and ensure the sustainable development of nearshore ecosystems, a comprehensive approach is essential to bolster fundamental research. This entails conducting systematic studies of marine ecosystems and their components to comprehend ecological behaviors, population dynamics, and relationships with environmental factors in order to uncover key triggers of biological outbreaks and make predictive assessments. Furthermore, fostering interdisciplinary collaboration to integrate knowledge from diverse fields such as marine biology, ecology, climatology, and environmental science will enhance comprehension of intricate ecosystems and facilitate the development of effective management strategies. Additionally, advancing new technologies and methodologies, including high-resolution remote sensing technology, genomics, and data analysis tools, will augment the capacity for ecosystem study while aiding scientists in identifying emerging ecological threats and opportunities. Building upon these studies supported by advanced technologies enables the reinforcement of comprehensive integrated research at an ecosystem level that can forecast future trends, thereby providing a scientific foundation and technical backing for the sustainable development of China’s nearshore ecosystems and ultimately promoting robust functioning of marine ecosystems while effectively addressing potential future marine biological disasters.

## 8. Conclusions

This review focuses on the current status of marine biological disasters, the fundamental biological background, and the causes of these outbreaks in China’s marine ecosystem. These outbreak species not only seriously damage fishery resources, tourism, and coastal industries but also pose a significant threat to the stability of the marine ecosystem, especially in sensitive areas such as nuclear power plants and mariculture areas. Existing studies show that marine biological outbreaks are the result of the combined effects of multiple factors. In this regard, we can further strengthen basic research, carry out resource utilization of outbreak organisms, formulate more scientific strategies for enhancement and release activities or mariculture, and adopt prevention and control measures with less damage to the marine ecology. However, the existing prevention and control strategies mostly focus on post-outbreak treatment, and future research and management should pay more attention to the early monitoring and prevention of disasters. Currently, the marine ecological disaster monitoring system in China only covers green tides and red tides with no inclusion of outbreaks of marine organisms such as Cnidaria (jellyfish organisms), Annelida (*U. unicinctus*), Mollusca (*P. kinglipini*), Arthropoda (*A. chinensis*), and Echinodermata (Asteroidea organisms, Ophiuroidea organisms, and *A. molpadioides*) within the standardized regulatory scope. It is recommended that more severe marine fauna disasters be gradually incorporated into China’s regulatory system for marine disasters based on observed trends in marine ecosystem changes and potential threats posed by outbreaks of marine fauna to the environment. This approach will bolster ecological protection efforts, mitigate economic losses, safeguard public health, and advance scientific research.

## Figures and Tables

**Table 1:** A list of the sea areas where China’s nuclear power plants are located and whether there have been outbreaks of marine faunas.

The Administrative Region Where the Nuclear Power Plant Is Located	The Name of Nuclear Power Plant	The Sea Area Where the Nuclear Power Plant Is Located	The Number of Generating Units in the Nuclear Power Plant	Whether a Biological Outbreak Disaster Exists
Fujian Province	Ningde Nuclear Power Plant	The East China Sea	4	√
Fujian Province	Fuqing Nuclear Power Plant	The East China Sea	6	-
Guangdong Province	Daya Bay Nuclear Power Plant	The South China Sea	2	√
Guangdong Province	Ling’ao Nuclear Power Plant	The South China Sea	4	√
Guangdong Province	Yangjiang Nuclear Power Plant	The South China Sea	6	√
Guangdong Province	Taishan Nuclear Power Plant	The South China Sea	2	-
Guangxi Zhuang Autonomous Region	Fangchenggang Nuclear Power Plant	The South China Sea	4	-
Hainan Province	Changjiang Nuclear Power Plant	The South China Sea	2	-
Jiangsu Province	Tianwan Nuclear Power Plant	The Yellow Sea	6	-
Liaoning Province	Hongyanhe Nuclear Power Plant	The Bohai Sea	6	√
Shandong Province	Haiyang Nuclear Power Plant	The Yellow Sea	2	-
Shandong Province	Shidao Bay Nuclear Power Plant	The Yellow Sea	1	-
Taiwan Province	The Third Nuclear Power Plant	The South China Sea	2	-
Zhejiang Province	Fangjiashan Nuclear Power Station	The East China Sea	2	-
Zhejiang Province	Sanmen Nuclear Power Plant	The East China Sea	2	-
Zhejiang Province	Qinshan Nuclear Power Plant	The East China Sea	1	-
Zhejiang Province	Qinshan Nuclear Power Plant Phase II	The East China Sea	4	-
Zhejiang Province	Qinshan Nuclear Power Plant Phase III	The East China Sea	2	-

Note: The statistical time node of the nuclear power plant that is currently operating is March 2024, and the statistical scope includes the Chinese Mainland (https://www.china-nea.cn/site/content/44813.html, access date: 15 July 2024), as well as Hong Kong Special Administrative Region, Macao Special Administrative Region, and Taiwan Province. Among them, ‘√’ indicates the occurrence of a marine organism outbreak at the nuclear power plant, while ‘-’ represents the current absence of an outbreak, but it does not imply whether there will be one in the future.
